# Effects of Platelet-Rich Plasma Dose and Application Strategy on Post-Thaw Spermatological Parameters in Goat Semen

**DOI:** 10.3390/vetsci13030245

**Published:** 2026-03-05

**Authors:** Ahmet Eser, Kemal Bağcı, Abdurrahman Alakuş, Aslıhan Çakır Cihangiroğlu, İkra Karaağaç, Selin Yağcıoğlu, Ramazan Arıcı, Kamber Demir

**Affiliations:** 1 Department of Reproduction and Artificial Insemination, Faculty of Veterinary Medicine, Siirt University, Siirt TR-56100, Türkiye; kemal.bagci@siirt.edu.tr (K.B.); aslihan.cakir@siirt.edu.tr (A.Ç.C.); 2 Department of Reproduction and Artificial Insemination, Institute of Health Sciences, Dicle University, Diyarbakır TR-21280, Türkiye; alakusabdurrahman29@gmail.com; 3 Department of Reproduction and Artificial Insemination, Graduate Education Institute, Istanbul University-Cerrahpaşa, Istanbul TR-34320, Türkiye; ikrakaraagac@ogr.iuc.edu.tr; 4 Department of Reproduction and Artificial Insemination, Faculty of Veterinary Medicine, Istanbul University-Cerrahpaşa, Istanbul TR-34320, Türkiye; selin.yagcioglu@iuc.edu.tr (S.Y.); ramazan.arici@iuc.edu.tr (R.A.); kamberdemir@iuc.edu.tr (K.D.)

**Keywords:** Boer goat, CASA, PRP, semen cryopreservation, sperm quality, flow cytometry

## Abstract

This study investigated the effects of different PRP doses and application strategies on sperm quality in goats during cryopreservation. Semen samples were collected weekly from six Boer goats and treated with PRP either by means of pre-incubation or supplementation of the semen extender at concentrations of 10, 20, and 40 × 10^6^ platelets/mL. Computer-assisted sperm analysis (CASA) was performed after equilibration and again after thawing to assess sperm motility parameters, including progressive motility. Post-thaw sperm functional integrity was further evaluated using flow cytometry, including assessments of plasma membrane integrity, acrosome integrity, high mitochondrial membrane potential (hMMP), sperm viability, oxidative stress, and DNA integrity. The results demonstrated that pre-incubation with PRP at 10 × 10^6^ platelets/mL (pre-PRP10) yielded the most pronounced improvements in total and progressive motility after thawing, as well as in plasma membrane integrity, viability, and mitochondrial activity, while significantly reducing oxidative stress and DNA fragmentation compared with other PRP doses and application strategies. Supplementation of the extender with PRP at 20 × 10^6^ platelets/mL also showed favorable effects on functional sperm parameters; however, it did not result in significant improvements in oxidative stress or DNA fragmentation. In contrast, the highest PRP dose (40 × 10^6^ platelets/mL) did not confer additional benefits. None of the PRP treatments improved post-thaw acrosome integrity. In conclusion, these findings highlight the importance of PRP dose optimization and direct sperm–PRP interaction, identifying pre-incubation with PRP at 10 × 10^6^ platelets/mL as the most effective approach for enhancing post-thaw sperm quality in goats.

## Simple Summary

Freezing goat semen is widely used in animal breeding, but the freezing and thawing process can seriously damage sperm cells, reducing their movement and overall quality. Platelet-rich plasma (PRP) is a natural biological product rich in growth factors and antioxidants, and it has recently attracted attention for its potential protective effects on sperm cells. In this study, we investigated whether adding PRP to goat semen before freezing could protect sperm quality after thawing. Semen samples from Boer goats were treated with different PRP doses (10, 20, and 40 million platelets per milliliter) using two different methods: direct pre-incubation of sperm with PRP and addition of PRP to the freezing solution. Sperm movement, cell membrane health, energy production, oxidative stress, and DNA damage were evaluated after thawing. The results showed that pre-incubating sperm with a low dose of PRP (10 million platelets/mL) before freezing provided the best protection. This treatment improved sperm movement, including progressive motility, preserved cell membranes and mitochondrial activity, and reduced oxidative stress and DNA damage. Higher PRP doses were not more effective. These findings suggest that using PRP in an optimized and carefully applied manner can help improve the success of goat breeding programs.

## 1. Introduction

Sperm cryopreservation is a well-established reproductive biotechnology that represents a critical component of animal breeding programs. Nevertheless, the freeze–thaw process induces substantial structural and functional damage to spermatozoa, including compromised motility and viability, plasma membrane disruption, diminished mitochondrial membrane potential, and DNA fragmentation [1]. The principal mechanisms underlying this damage include cold shock, intracellular ice crystal formation, osmotic stress, and oxidative insult occurring throughout the cryopreservation process [1,2]. Consequently, researchers have focused on optimizing cryopreservation protocols and developing protective media formulations to mitigate freeze–thaw-induced sperm damage [3]. Notably, substantial advances have emerged from investigations examining the cryoprotective efficacy of supplemental additives, including peptides, fatty acids, animal sera, nanoparticles, plant-derived oils, bioactive compounds, and antioxidants, which confer protection through diverse molecular mechanisms [4,5,6,7,8].

Platelet-rich plasma (PRP) is a blood-derived product characterized by platelet concentrations three- to seven-fold higher than physiological levels in whole blood [9,10]. Due to its diverse complement of bioactive molecules, PRP has been widely employed in regenerative medicine applications across dermatology, orthopedics, and dentistry [9]. These bioactive constituents include growth factors such as transforming growth factor beta (TGF-β), fibroblast growth factor (FGF), vascular endothelial growth factor (VEGF), insulin-like growth factor 1 (IGF-1), platelet-derived growth factor (PDGF), and nerve growth factor (NGF), as well as antioxidant enzymes (superoxide dismutase, SOD), essential minerals (zinc, Zn; calcium, Ca), neurotransmitters (histamine, serotonin), and energy metabolites (adenosine triphosphate, ATP) [11,12,13,14,15].

Studies investigating the effects of PRP constituents on sperm cryopreservation have demonstrated multiple beneficial outcomes. Research has shown that TGF-β and VEGF enhance sperm motility [16,17,18], while zinc and calcium ions play pivotal roles in sperm capacitation and the acrosome reaction [19,20]. Moreover, IGF-1, NGF, ATP, zinc, and SOD have been reported to protect spermatozoa against cold shock-induced damage and improve post-thaw sperm quality [21,22,23,24,25]. Fibroblast growth factor has been shown to positively influence progressive sperm motility through activation of extracellular signal-regulated kinase and protein kinase B signaling pathways [26,27], whereas serotonin enhances sperm kinetic parameters, particularly curvilinear velocity [28]. Collectively, these findings underscore the potential of PRP components to enhance sperm cryopreservation outcomes.

Research has demonstrated that incorporating PRP into semen cryopreservation media serves as an effective strategy to minimize structural and functional damage to sperm during the freeze–thaw process. This protective effect is attributed to the diverse biological agents present in PRP, which exhibit cryoprotective properties. The efficacy of this approach has been validated across multiple species, suggesting its broad applicability in reproductive biotechnology [29].

Previous studies have established that a PRP concentration of 20 × 10^6^ platelets/mL is effective in goat sperm cryopreservation; however, the potential benefits of higher concentrations remain to be fully elucidated [8]. In addition, Yan et al. [10] emphasized the need for systematic evaluation of alternative PRP application strategies, including pre-cryopreservation treatment and post-thaw supplementation. Accumulating evidence further indicates that in vitro pre-incubation of spermatozoa with PRP for different durations exerts a beneficial effect on key sperm quality parameters [30,31,32].

Accordingly, the present study was designed to systematically evaluate, for the first time, the effects of platelet-rich plasma applied at different concentrations (10 × 10^6^/mL, 20 × 10^6^/mL, and 40 × 10^6^/mL) using distinct application strategies (extender supplementation and pre-incubation) on post-thaw spermatological parameters in Boer goat semen. We hypothesized that PRP application would enhance post-thaw sperm quality in a dose- and method-dependent manner.

## 2. Materials and Methods

The study was carried out during the breeding season at the Small Ruminant Reproductive Biotechnology Research Center in Siirt, Türkiye. All Boer goats (n = 6) were maintained under standardized management conditions, with ad libitum access to clean drinking water and natural photoperiod. The goats were provided with a balanced diet comprising alfalfa hay and a commercial concentrate formulated to meet the nutritional requirements for maintenance and reproductive performance.

All procedures involving animals were conducted in accordance with the ethical principles of animal experimentation and were approved by the Local Ethics Committee for Animal Research at Siirt University, Türkiye (30.01.2024–2024/06).

### 2.1. Preparation of Platelet-Rich Plasma

PRP was prepared using a double-centrifugation method. Whole blood specimens (20–30 mL) were obtained aseptically from caprine subjects and transferred into sterile anticoagulant-containing tubes, then centrifuged at 160× *g* for 20 min. The supernatant representing the upper two-thirds of the platelet-poor plasma fraction was subsequently removed using a sterile syringe and discarded. The remaining lower third was then transferred into sterile anticoagulant-free tubes and subjected to a second centrifugation at 400× *g* for 15 min. After the second centrifugation, the upper two-thirds of the platelet-poor plasma was removed, and the final lower third (approximately 3–4 mL) was collected as PRP [33]. Platelet enumeration in the obtained PRP was conducted using an automated hematology analyzer (BC-60R Vet, Mindray, Shenzhen, China). The PRP platelet concentration was 1.649 × 10^9^ platelets/L. PRP activation was achieved by supplementation with 10% calcium chloride [8].

### 2.2. Experimental Design

**Figure 1:**
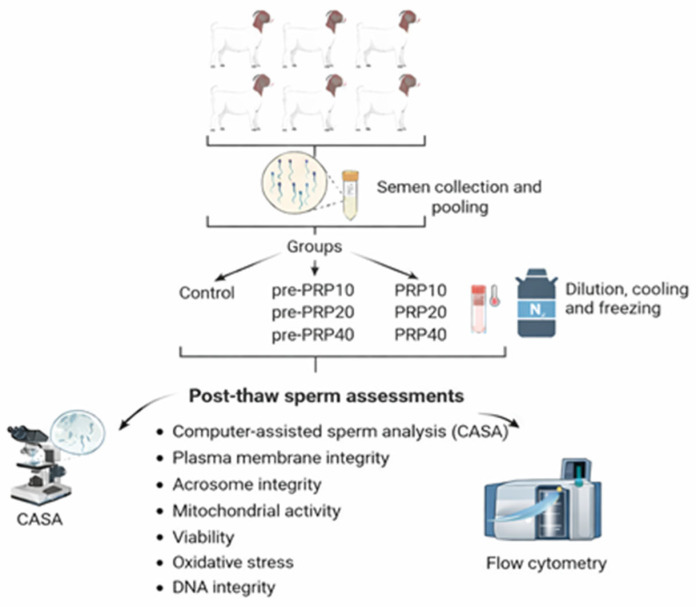
The graphical presentation of the study.

The experimental design of the study is presented in Figure 1. The study comprises seven groups in total: one control group and six experimental groups.

1. Control: PRP-free extender
2. Pre-PRP10: 10 × 10^6^ platelets/mL PRP (15-min pre-incubation with spermatozoa)
3. Pre-PRP20: 20 × 10^6^ platelets/mL PRP (15-min pre-incubation with spermatozoa)
4. Pre-PRP40: 40 × 10^6^ platelets/mL PRP (15-min pre-incubation with spermatozoa)
5. PRP10: 10 × 10^6^ platelets/mL PRP (supplemented to extender)
6. PRP20: 20 × 10^6^ platelets/mL PRP (supplemented to extender)
7. PRP40: 40 × 10^6^ platelets/mL PRP (supplemented to extender)

### 2.3. Semen Collection and Evaluation of Individual Spermatological Characteristics

Semen samples were collected weekly from six Boer goats throughout the breeding season, yielding seven replicate ejaculates per individual (n = 7). Collections were performed by means of electroejaculation using a commercial electroejaculator (e320, Minitube, Tiefenbach, Germany) following the protocol described by Ungerfeld et al. [34].

Immediately post-collection, ejaculates were maintained at 26 °C in a water bath and subjected to comprehensive spermatological evaluation. Ejaculate volume was determined using graduated conical tubes with 0.1 mL precision. Sperm concentration was assessed photometrically using a species-specific analyzer (Ovine-Caprine AccuRead, IMV Technologies, L’Aigle, France). Mass activity was examined via phase-contrast microscopy (Eclipse Ci-L, Nikon, Tokyo, Japan) at 5× magnification. Wave motion intensity at the periphery of semen droplets was scored using a 4-point scale (0–4), where higher scores represented more vigorous wave-like patterns. Individual sperm motility parameters were assessed using computer-assisted sperm analysis (CASA). Ejaculates were selected for cryopreservation only when fulfilling the following minimum quality thresholds: sperm concentration ≥ 1.0 × 10^9^ spermatozoa/mL, mass motility score ≥ 3, and total motility ≥ 80%.

### 2.4. Semen Cryopreservation

Collected ejaculates were pooled to minimize inter-individual variability, and standardized aliquots containing 400 × 10^6^ spermatozoa were distributed into 15 mL centrifuge tubes assigned to respective experimental groups. A commercially available cryoprotectant-supplemented extender (Andromed^®^; Minitube GmbH, Tiefenbach, Germany) was used for dilution of all experimental samples.

The control group was diluted stepwise with PRP-free extender at concentrations of 10%, 20%, 30%, and 40% at 8-min intervals. The PRP10, PRP20, and PRP40 groups were subjected to identical stepwise dilution protocols using extender supplemented with the corresponding PRP concentration.

For the PRP pre-incubation experimental groups (pre-PRP10, pre-PRP20, pre-PRP40), sperm samples were initially incubated with the corresponding concentrations of platelet-rich plasma for 15 min at 26 °C. Following pre-incubation, samples underwent stepwise dilution with commercial extender at sequential concentrations of 10%, 20%, 30%, and 40% at 8-min intervals.

Following dilution, samples were subjected to controlled cooling to 5 °C and equilibrated at this temperature for 2 h. Following the equilibration period, sperm motility was evaluated using CASA. Equilibrated semen was subsequently loaded into 0.25 mL French straws (MPP Uno, Minitube, Tiefenbach, Germany) and positioned in an automated programmable freezing apparatus (Icecube 14S, Sylab, Vienna, Austria). The cryopreservation protocol utilized a two-stage freezing curve [35]: samples were cooled from 5 °C to −8 °C at a rate of 3 °C/min, followed by rapid cooling from −8 °C to −120 °C at 15 °C/min. Upon completion of the freezing cycle, cryopreserved straws were promptly transferred to liquid nitrogen storage containers and maintained at −196 °C until thawed for analysis.

### 2.5. Post-Thaw Sperm Analysis

For post-thaw evaluation, straws were thawed in a water bath at 37 °C for 30 s prior to assessment of sperm characteristics.

#### 2.5.1. Assessment of Sperm Motility and Kinematics

Sperm motility and kinematic parameters were assessed using a computer-assisted sperm analysis (CASA) system (Sperm Class Analyzer v3.2.0, Microptics S.L., Barcelona, Spain) equipped with a high-speed camera (Basler ACA1300-200UC, Basler Vision Technologies, Ahrensburg, Germany) operating at 60 frames/s. The system was calibrated for caprine spermatozoa following manufacturer specifications.

The following parameters were evaluated: total motility (MOT, %), progressive motility (pMOT, %), mean path velocity (VAP, µm/s), straight-line velocity (VSL, µm/s), curvilinear velocity (VCL, µm/s), amplitude of lateral head displacement (ALH, µm), beat cross frequency (BCF, Hz), straightness (STR, VSL/VAP × 100, %), linearity (LIN, VSL/VCL × 100, %), and wobble (WOB, VAP/VCL × 100, %).

CASA settings included brightness 60, contrast 750, and light intensity 1000. Motility thresholds were VCL > 80 µm/s, VSL > 50 µm/s, and VAP > 25 µm/s. Spermatozoa with STR > 80% were classified as progressively motile [36].

For each analysis, 3 µL aliquots were loaded onto pre-warmed slides (37 °C), covered with 22 × 22 mm coverslips, and examined by means of phase-contrast microscopy (Eclipse Ci-L, Nikon, Tokyo, Japan) using a 10× negative phase-contrast objective at 37 °C. At least three fields per sample were analyzed, evaluating a minimum of 600 spermatozoa per sample.

#### 2.5.2. Flow Cytometry Analyses

Sperm plasma membrane integrity, acrosome integrity, mitochondrial membrane potential, viability, oxidative stress, and DNA integrity were evaluated using flow cytometry (Guava easyCyte™, Luminex Corporation, Austin, TX, USA). A minimum of 10,000 events per sample were analyzed to ensure statistical reliability.

##### Assessment of Sperm Plasma Membrane Integrity

The proportion of viable spermatozoa with intact plasma membranes was assessed using a dual fluorescent staining protocol as described by Câmara et al. [37]. Thawed samples were diluted to a concentration of 50 × 10^6^ spermatozoa/mL in Tris-based solution. A 100 µL aliquot of each sample was incubated with 0.5 µL carboxyfluorescein diacetate (CFDA; 0.46 mg/mL; Cat. no. C5041, Sigma-Aldrich, St. Louis, MO, USA) and 0.5 µL propidium iodide (PI; 0.5 mg/mL; Cat. no. 81845, Sigma-Aldrich), followed by the addition of 200 µL Tris solution. Samples were incubated in the dark at room temperature for 10 min, transferred to 96-well plates, and subsequently analyzed by means of flow cytometry. CFDA fluorescence was detected at 488 nm excitation/517 nm emission (green), whereas PI fluorescence was detected at 488 nm excitation/617 nm emission (red). Spermatozoa exhibiting green fluorescence without red fluorescence (CFDA^+^/PI^−^) were classified as viable cells with intact plasma membranes.

##### Assessment of Sperm Acrosome Integrity

The proportion of viable spermatozoa with intact acrosomes was evaluated using a dual-staining protocol with fluorescein isothiocyanate-conjugated peanut agglutinin (FITC-PNA; Cat. no. L7381, Sigma-Aldrich, St. Louis, MO, USA) and propidium iodide (PI; Cat. no. 81845, Sigma-Aldrich) as described by Marco-Jiménez et al. [38]. Thawed semen was diluted to a concentration of 1 × 10^6^ spermatozoa/mL in Tris buffer, centrifuged at 700× *g* for 5 min, and the resulting pellet was resuspended in 1000 µL Tris buffer. Subsequently, a 100 µL aliquot of the sample was mixed with 2 µL FITC-PNA (100 µg/mL), 2 µL PI (0.5 mg/mL), and 200 µL Tris buffer. Samples were incubated in the dark at room temperature for 10 min, and 300 µL was transferred to 96-well plates for flow cytometric analysis. FITC-PNA fluorescence was measured at 488 nm excitation/525 nm emission (green), whereas PI fluorescence was measured at 488 nm excitation/617 nm emission (red). Spermatozoa exhibiting intact acrosomes were classified as FITC-PNA^−^/PI^−^.

##### Assessment of Sperm Mitochondrial Membrane Potential

Sperm mitochondrial membrane potential (MMP) was assessed using the lipophilic cationic dye 5,5′,6,6′-tetrachloro-1,1′,3,3′-tetraethylbenzimidazolylcarbocyanine iodide (JC-1; Cat. no. T4069, Sigma-Aldrich, St. Louis, MO, USA) as described by Dariush et al. [39] with modifications. Thawed semen samples were diluted to a concentration of 50 × 10^6^ spermatozoa/mL in Tris-based buffer. A 100 µL aliquot of the diluted sample was incubated with 0.5 µL JC-1 stock solution (3 mM in DMSO) and 200 µL Tris buffer at 38 °C for 40 min in the dark. Following incubation, samples were transferred to 96-well microplates for flow cytometric analysis. JC-1 fluorescence was detected using dual emission channels: orange–red fluorescence (excitation 488 nm/emission 590 nm) representing J-aggregates in mitochondria with high membrane potential, and green fluorescence (excitation 488 nm/emission 525 nm) representing JC-1 monomers in mitochondria with low membrane potential. Spermatozoa exhibiting high orange–red fluorescence were classified as possessing high mitochondrial membrane potential (hMMP).

##### Assessment of Sperm Viability

Cell viability was determined using the Zombie Green™ Fixable Viability Kit (Cat. no. 423112, BioLegend, San Diego, CA, USA) according to the manufacturer’s protocol with minor modifications [40]. The fluorescent dye was reconstituted by adding 100 µL DMSO to one vial of lyophilized Zombie Green reagent and mixing until completely dissolved. Prior to staining, samples were washed once with phosphate-buffered saline (PBS, pH 7.4) by centrifugation at 400× *g* for 5 min at room temperature. The resulting pellets were resuspended in PBS to achieve a final concentration of 1 × 10^6^ spermatozoa/mL. Subsequently, 1 µL of reconstituted Zombie Green dye was added to a 100 µL aliquot of each sample. Samples were incubated at room temperature in the dark for 30 min, transferred to 96-well plates, and subsequently analyzed by means of flow cytometry. Zombie Green fluorescence was measured at 488 nm excitation/525 nm emission (green). Viable spermatozoa exhibited minimal green fluorescence and were classified as Zombie Green^−^, whereas dead spermatozoa with compromised membranes exhibited intense green fluorescence and were classified as Zombie Green^+^.

##### Quantification of Intracellular Superoxide Levels in Spermatozoa

Oxidative stress in post-thaw spermatozoa was evaluated by quantifying intracellular superoxide radical levels using the Muse^®^ Oxidative Stress Kit (Cat. no. MCH100111, Luminex Corporation, Austin, TX, USA) following the manufacturer’s protocol. The assay employs dihydroethidium (DHE) as a fluorogenic probe for reactive oxygen species (ROS) detection. Semen samples were first diluted to 100 × 10^6^ spermatozoa/mL in assay buffer. A 10 µL aliquot of each diluted sample was then combined with 190 µL Muse^®^ Oxidative Stress reagent containing DHE and incubated at 37 °C under dark conditions for 30 min. Following incubation, the stained samples were transferred to 96-well plates for flow cytometric analysis. Upon oxidation, DHE exhibits red fluorescence, which was measured at 488 nm excitation/585–605 nm emission. Spermatozoa displaying red fluorescence intensity above the threshold were designated as ROS-positive cells, reflecting elevated superoxide production and oxidative stress [41].

##### Sperm Chromatin Structure Assay (SCSA)

Deoxyribonucleic acid (DNA) integrity was assessed using the sperm chromatin structure assay (SCSA) as described by Evenson et al. [42]. Semen samples were diluted with TNE buffer (0.15 M NaCl, 0.01 M Tris-HCl, 1 mM disodium EDTA, pH 7.4) to achieve a final concentration of 2 × 10^6^ spermatozoa/mL. All reagents were maintained at 4 °C prior to use. Samples were treated with 400 µL acid-detergent solution (0.08 N HCl, 0.15 M NaCl, 0.1% Triton X-100, pH 1.2) at 4 °C for approximately 30 s to induce DNA denaturation in situ. Subsequently, 1.20 mL acridine orange staining solution (0.037 M citric acid, 0.126 M Na_2_HPO_4_, 0.0011 M disodium EDTA, 0.15 M NaCl, 6 µg/mL acridine orange, pH 6.0) was immediately added to each sample. Flow cytometric analysis was subsequently performed to distinguish DNA integrity based on fluorescence patterns. Spermatozoa harboring intact double-stranded DNA (dsDNA) emitted green fluorescence at 488 nm excitation/515–530 nm emission, while those containing denatured single-stranded DNA (ssDNA) emitted red fluorescence at 488 nm excitation/630–640 nm emission.

### 2.6. Statistical Analysis

Statistical analyses were performed using SPSS Statistics software version 22.0 (IBM Corporation, Armonk, NY, USA). Prior to analysis, all spermatological parameters were assessed for normality using the Shapiro–Wilk test and for homogeneity of variance using Levene’s test. Differences in spermatological parameters among experimental groups were evaluated using one-way analysis of variance (ANOVA). When significant differences were detected, post hoc pairwise comparisons were conducted using Duncan’s multiple range test. Data are presented as mean ± standard error of the mean (SEM). Statistical significance was set at *p* < 0.05 for all analyses.

## 3. Results

Post-equilibration CASA analysis revealed no significant differences among the experimental groups in terms of total motility, progressive motility, VAP, VSL, VCL, ALH, BCF, STR, or LIN (*p* > 0.05). In contrast, WOB values were significantly higher in the pre-PRP10 and PRP10 groups (10 × 10^6^ platelets/mL) compared with the control group (*p* < 0.05), while no significant differences were observed among the PRP-treated groups (*p* > 0.05). Post-equilibration CASA parameters are presented in Table 1.

Post-thaw CASA analysis indicated that the pre-PRP10 group exhibited the highest total motility, with values significantly greater than those observed in the control, pre-PRP40, and PRP40 groups (*p* < 0.05). Progressive motility demonstrated a similar trend, as the pre-PRP10 group showed significantly higher values compared with the control and PRP40 groups (*p* < 0.05), whereas no significant differences were detected among the remaining groups (*p* > 0.05). Likewise, VSL values were significantly elevated in the pre-PRP10 group relative to the control and PRP40 groups (*p* < 0.05). In contrast, no significant differences were observed among the groups for VAP, VCL, ALH, BCF, STR, LIN, or WOB parameters (*p* > 0.05). The post-thaw spermatological parameters are presented in Table 2.

Flow cytometry-derived spermatological parameters are presented in Table 3. Sperm plasma membrane integrity was significantly higher in the pre-PRP10 group compared to the control, pre-PRP40, PRP10, and PRP40 groups (*p* < 0.05). Conversely, acrosome integrity rates were comparable across all groups (*p* > 0.05). Mitochondrial membrane potential was significantly elevated in the pre-PRP10 and PRP20 groups relative to the control and PRP40 groups (*p* < 0.05), with no significant differences detected among the remaining groups (*p* > 0.05). Intracellular superoxide levels, assessed as an oxidative stress marker, were significantly lower in the pre-PRP10 group compared to the control and all other experimental groups (*p* < 0.05). Consistent with these findings, the pre-PRP10 group demonstrated significantly reduced DNA fragmentation compared to the control group and the other treatments (*p* < 0.05) except pre-PRP20, further confirming its superior cryoprotective efficacy.

## 4. Discussion

Semen cryopreservation remains essential for genetic improvement and germplasm conservation in caprine breeding programs; however, freeze–thaw-induced damage continues to limit its efficacy. Goat spermatozoa exhibit heightened susceptibility to cryoinjury, manifesting as reduced motility and viability, plasma membrane disruption due to species-specific membrane lipid composition, and oxidative stress-mediated functional and molecular alterations [43,44]. Consequently, optimization of caprine semen cryopreservation requires a multifactorial approach encompassing extender formulation, precise control of cooling-freezing-thawing kinetics, and consideration of contextual variables such as season, collection methodology, and breed-specific characteristics [43,44,45,46].

The application of PRP in sperm cryopreservation has gained increasing research attention in recent years, driven by the premise that freeze–thaw processes induce substantial cellular damage and that supplementation with bioactive, autologous components may mitigate cryoinjury while enhancing post-thaw sperm function [47,48,49]. PRP is characterized by elevated concentrations of platelets and their associated bioactive cargo, including growth factors, cytokines, and extracellular vesicles, which modulate the extracellular microenvironment to support cellular resilience under stress conditions—a mechanism that provides the rationale for its use as a cryopreservation supplement [50,51]. Within the context of livestock semen preservation, emerging evidence has investigated PRP’s potential to address species-specific and membrane-specific vulnerabilities that render spermatozoa particularly susceptible to cryodamage, thereby compromising post-thaw quality and fertility outcomes in assisted reproduction applications [48,52].

Current literature describes two principal methodological approaches for incorporating PRP into semen cryopreservation protocols: direct supplementation of PRP into the cryopreservation extender prior to the freeze–thaw process, and in vivo PRP administration aimed at enhancing endogenous sperm quality before collection, followed by conventional cryopreservation [29]. Against this backdrop, the present study was designed to systematically evaluate the effects of PRP applied at varying concentrations using two distinct application strategies—extender supplementation and pre-incubation—on post-thaw spermatological parameters in goat semen.

Sperm motility is universally recognized as one of the most critical determinants of male fertility, serving as a fundamental prerequisite for successful fertilization [53]. The assessment of sperm motility has evolved significantly from subjective manual microscopy—characterized by substantial inter-observer variability and poor reproducibility [54]—to sophisticated CASA systems that provide objective, quantitative measurements of motility and multiple kinematic parameters [55,56]. These parameters collectively characterize sperm movement quality and vigor, essential attributes for traversing the female reproductive tract and achieving oocyte penetration [57]. Across diverse species and experimental protocols, PRP supplementation has consistently been shown to improve both total and progressive motility, with reported gains ranging from approximately 12–20% in fresh semen following incubation to 30–40% in cryopreserved samples, depending on species and methodological approaches [29]. In the present study, PRP supplementation—either added directly to the extender or applied via pre-incubation prior to dilution—did not significantly affect post-equilibration CASA parameters in goat semen, with the exception of a modest improvement in WOB values at lower concentrations. In contrast, the beneficial effects of PRP became markedly more evident following thawing. Specifically, pre-incubation with PRP at the lowest concentration (10 × 10^6^ platelets/mL) significantly enhanced MOT, pMOT, and VSL values (*p* < 0.05). These motility findings are consistent with those reported by Alcay et al. [8], who investigated Saanen goats during the non-breeding season and observed that PRP supplementation at 10 × 10^6^ and 20 × 10^6^ platelets/mL improved post-thaw motility parameters. Similarly, Salama et al. [47] reported enhanced motility parameters following dilution, equilibration, and thawing in buck semen supplemented with PRP.

In other species, including buffalo bulls [48,52,58], rams [59], and humans [26], supplementation of semen extenders with PRP at various concentrations has been reported to improve sperm motility parameters. Similarly, studies in humans have demonstrated that PRP incubation at different durations and doses enhances sperm motility [60,61]. Conversely, Quintana et al. [62] reported that incubation with plasma rich in growth factors at concentrations of 5%, 10%, 20%, and 40% did not exert significant effects on motility parameters in human spermatozoa, while in horses, platelet-rich plasma lysate was shown to induce sperm agglutination, thereby negatively affecting motility. Within the existing literature, some studies have reported findings consistent with the present study, indicating that increasing PRP concentrations may adversely affect CASA-derived motility parameters [10,60,63,64], whereas others have shown that motility parameters are not influenced in a dose-dependent manner [8,31,32,59]. The discrepancies observed among studies may be attributed to differences in PRP concentrations and application strategies, PRP preparation protocols, cryopreservation techniques, and species-specific variations.

Flow cytometry was used to objectively evaluate post-thaw sperm functional parameters and cryopreservation-induced damage [65]. Combined with CASA, it provided a comprehensive assessment of semen quality [66].

PRP exerts membrane-protective effects through multiple interconnected mechanisms. First, PRP supplementation markedly attenuates oxidative stress in spermatozoa, with reported reductions in ROS levels of up to 38%, thereby limiting lipid peroxidation—a major contributor to cryopreservation-induced membrane damage—and preserving membrane fluidity and integrity [67]. Second, growth factors contained in PRP, including PDGF, TGF-β, and FGF, play critical roles in cellular repair processes, maintenance of membrane structural integrity, and inhibition of apoptotic signaling pathways that lead to membrane destabilization [18]. Third, PRP contributes to the preservation of mitochondrial membrane potential, an early marker of apoptosis that is positively associated with sperm motility, thereby indirectly supporting plasma membrane integrity through sustained ATP production and cellular energy homeostasis [67,68]. Consistent with these mechanisms and previous studies [8,10,47,58,59,64], flow cytometric analysis in the present study demonstrated that post-thaw plasma membrane integrity was significantly preserved in goat spermatozoa supplemented with PRP at a concentration of 20 × 10^6^ platelets/mL in the extender (*p* < 0.05), as well as in those pre-incubated with PRP at concentrations of 10 × 10^6^ and 20 × 10^6^ platelets/mL (*p* < 0.05).

In line with the findings related to motility and plasma membrane integrity, sperm viability and mitochondrial activity were also more favorably preserved in samples supplemented with PRP at 20 × 10^6^ platelets/mL in the extender (*p* < 0.05) and in those pre-incubated with PRP at 10 × 10^6^ platelets/mL (*p* < 0.05). Furthermore, post-thaw oxidative stress levels and DNA fragmentation rates were markedly reduced in the pre-PRP10 group compared with the control and other PRP dose and application strategies (*p* < 0.05), indicating a superior protective effect. These observations are supported by previous studies reporting that PRP reduces oxidative stress through activation of the Nrf2 signaling pathway and exhibits pronounced antioxidant and anti-apoptotic properties [26,31,60]. In addition, PRP has been shown to enhance membrane and DNA stability via bioactive factors such as IGF-1, NGF, VEGF, and serotonin, thereby supporting sperm viability [10], while IGF-1 in particular has been reported to inhibit mitochondrial cytochrome c release, contributing to the preservation of mitochondrial function and cellular survival [60].

Although PRP supplementation improved several post-thaw sperm parameters, particularly in the pre-PRP10 and PRP20 groups, the higher concentration did not confer additional benefits compared to the control group. This observation is consistent with the findings of Soltani et al. [69]. The lack of further improvement at elevated doses may be attributed to receptor saturation or alterations in the biochemical and osmotic balance of the extender. Specifically, increased levels of amino acids in high-concentration PRP [47] may elevate osmotic pressure and induce hypertonicity, thereby compromising sperm membrane stability and motility during cryopreservation [70]. Although some studies have reported beneficial effects of homologous PRP even at higher concentrations [27], our results indicate that maximal cryoprotective efficacy was achieved at a lower dose. Importantly, the higher concentration was not associated with detrimental effects, as no significant deterioration in sperm quality parameters was detected compared to the control group. Collectively, these findings suggest that even with homologous PRP, its biological effects are dose-dependent and require careful optimization to ensure maximal efficacy [60,69].

Previous studies in goats have demonstrated that PRP supplementation of semen extenders can improve plasma membrane integrity, acrosome integrity, mitochondrial membrane potential, and DNA integrity [8,47]. However, in the present study, extender-based PRP supplementation did not significantly enhance post-thaw oxidative stress parameters or DNA integrity, nor did any dose or application strategy improve acrosome integrity. Such discrepancies may stem from differences in extender composition, PRP dosing strategies, and methodological variations across studies. Notably, divergent results have been reported even within the same breed [47], emphasizing the critical role of PRP preparation and application protocols. In our study, PRP dosage was standardized according to platelet concentration (platelets/mL), whereas other investigations have administered PRP as a percentage of semen volume. These methodological differences likely contribute to variability in outcomes and underscore the need for standardized PRP preparation and application protocols to improve reproducibility and enable reliable inter-study comparisons.

## 5. Conclusions

In conclusion, the present study demonstrates that pre-incubation with PRP at 10 × 10^6^ platelets/mL represents the most effective strategy for improving post-thaw sperm quality in Boer goats, particularly through its protective effects against oxidative stress and DNA fragmentation. The pre-PRP10 group exhibited superior preservation of functional and structural sperm parameters, suggesting that early exposure to PRP-derived bioactive factors enhances cellular resilience prior to cryopreservation. This protective effect is likely mediated by antioxidant activity and growth factor–induced stabilization of membrane integrity and chromatin structure before the onset of cryo-induced stress. While extender supplementation with PRP at 20 × 10^6^ platelets/mL also yielded favorable outcomes, its effects were less pronounced in terms of oxidative balance and DNA integrity. Overall, these findings underscore the critical importance of direct sperm–PRP interaction and dose optimization, identifying pre-incubation with PRP at 10 × 10^6^ platelets/mL as a promising approach for mitigating oxidative damage and preserving genomic integrity during sperm cryopreservation. Future studies should aim to standardize PRP preparation and application protocols and further elucidate the molecular mechanisms underlying its antioxidative and cryoprotective effects.

## Figures and Tables

**Table 1:** Effect of PRP treatments on CASA parameters (mean ± SEM) after equilibration.

CASA	Treatment						
	Control	Pre-PRP10	Pre-PRP20	Pre-PRP40	PRP10	PRP20	PRP40
MOT	82.19 ± 3.81	83.80 ± 3.67	84.17 ± 3.88	81.71 ± 5.17	87.29 ± 2.88	86.31 ± 2.46	87.03 ± 2.63
pMOT	20.73 ± 2.56	25.64 ± 4.58	23.68 ± 3.83	19.89 ± 2.69	26.55 ± 5.04	24.78 ± 5.32	20.60 ± 2.12
VAP	96.86 ± 9.60	101.80 ± 6.20	102.07 ± 4.57	92.20 ± 4.05	102.11 ± 5.77	101.82 ± 4.03	100.67 ± 5.01
VSL	51.93 ± 4.78	61.63 ± 6.84	56.48 ± 5.04	50.14 ± 3.30	59.37 ± 6.07	58.24 ± 5.38	54.38 ± 3.53
VCL	191.41 ± 20.17	192.18 ± 11.83	199.64 ± 9.48	181.02 ± 10.78	194.65 ± 12.97	198.37 ± 6.22	196.32 ± 11.98
ALH	4.40 ± 0.39	4.26 ± 0.28	4.52 ± 0.21	4.23 ± 0.30	4.42 ± 0.33	4.52 ± 0.17	4.54 ± 0.28
BCF	15.52 ± 1.70	17.78 ± 1.30	17.28 ± 0.97	15.43 ± 0.93	16.83 ± 1.27	17.03 ± 1.36	16.56 ± 1.12
STR	51.81 ± 1.78	57.84 ± 3.23	54.87 ± 2.90	53.43 ± 2.79	56.76 ± 3.95	55.34 ± 3.36	53.62 ± 1.50
LIN	23.47 ± 4.12	33.12 ± 2.96	29.05 ± 2.69	29.59 ± 2.36	32.57 ± 3.79	30.43 ± 2.51	29.79 ± 1.40
WOB	45.69 ± 4.32 ^b^	53.21 ± 1.55 ^a^	51.27 ± 1.58 ^a,b^	51.56 ± 1.33 ^a,b^	53.02 ± 2.40 ^a^	51.09 ± 1.48 ^a,b^	51.88 ± 1.12 ^a,b^

Different literals within lines mean statistical differences, *p* < 0.05. MOT, total motility (%); pMOT, progressive motility (%); VAP, average path velocity (µm/s); VSL, straight-linear velocity (µm/s); VCL, curvilinear velocity (µm/s); ALH, amplitude of lateral head displacement (µm); BCF, beat cross frequency (Hz); STR, straightness (%); LIN, movement linearity (%); WOB, wobble (%).

**Table 2:** Influence of different PRP doses and application methods on post-thaw CASA parameters (mean ± SEM).

CASA	Treatment						
	Control	Pre-PRP10	Pre-PRP20	Pre-PRP40	PRP10	PRP20	PRP40
MOT	59.53 ± 6.91 ^c^	83.34 ± 2.72 ^a^	69.19 ± 5.70 ^a,b,c^	66.35 ± 7.37 ^b,c^	78.93 ± 2.79 ^a,b^	79.75 ± 3.62 ^a,b^	58.44 ± 6.18 ^c^
pMOT	22.93 ± 4.47 ^b^	37.26 ± 3.06 ^a^	30.78 ± 5.56 ^a,b^	27.79 ± 4.52 ^a,b^	33.69 ± 3.52 ^a,b^	35.24 ± 1.99 ^a,b^	22.98 ± 3.84 ^b^
VAP	50.28 ± 4.97	63.95 ± 4.07	59.38 ± 6.59	53.60 ± 4.75	56.67 ± 2.01	59.43 ± 5.34	50.73 ± 2.46
VSL	34.23 ± 3.76 ^b^	47.44 ± 5.05 ^a^	44.52 ± 5.90 ^a,b^	38.51 ± 3.78 ^a,b^	39.89 ± 1.62 ^a,b^	42.52 ± 3.85 ^a,b^	34.17 ± 2.67 ^b^
VCL	96.87 ± 9.32	112.71 ± 4.25	106.31 ± 11.11	101.24 ± 8.11	105.05 ± 5.53	108.85 ± 12.39	81.33 ± 14.21
ALH	2.55 ± 0.11	2.51 ± 0.07	2.41 ± 0.18	2.39 ± 0.16	2.49 ± 0.16	2.53 ± 0.27	2.33 ± 0.10
BCF	10.92 ± 1.21	13.19 ± 0.63	12.86 ± 1.43	12.10 ± 1.25	12.73 ± 0.68	13.19 ± 0.99	10.27 ± 0.90
STR	61.09 ± 2.14	66.85 ± 2.43	66.63 ± 2.60	63.13 ± 2.03	65.22 ± 1.76	65.38 ± 1.71	61.32 ± 1.94
LIN	37.82 ± 2.46	43.65 ± 2.88	44.21 ± 3.11	37.96 ± 2.54	41.81 ± 2.08	42.19 ± 2.59	40.42 ± 2.20
WOB	55.77 ± 1.95	60.08 ± 1.94	60.89 ± 2.70	55.42 ± 1.92	59.19 ± 1.60	59.40 ± 2.40	58.85 ± 1.70

Different literals within lines mean statistical differences, *p* < 0.05. MOT, total motility (%); pMOT, progressive motility (%); VAP, average path velocity (µm/s); VSL, straight-linear velocity (µm/s); VCL, curvilinear velocity (µm/s); ALH, amplitude of lateral head displacement (µm); BCF, beat cross frequency (Hz); STR, straightness (%); LIN, movement linearity (%); WOB, wobble (%).

**Table 3:** Outcomes of different doses and application methods on post-thaw spermatological characteristics (mean ± SEM) assessed by means of flow cytometry.

Parameters (%)	Treatment						
	Control	Pre-PRP10	Pre-PRP20	Pre-PRP40	PRP10	PRP20	PRP40
Plasma membrane integrity	37.71 ± 2.95 ^cd^	54.50 ± 2.24 ^a^	46.65 ± 2.58 ^a,b^	42.86 ± 2.50 ^b,c,d^	45.60 ± 3.29 ^b,c^	51.09 ± 2.31^a,b^	36.86 ± 3.05 ^d^
Acrosome integrity	39.74 ± 4.54	47.16 ± 3.34	44.41 ± 2.44	44.42 ± 3.08	41.32 ± 2.43	41.32 ± 2.70	44.09 ± 2.38
hMMP	61.77 ± 1.32 ^b^	68.03 ± 1.29 ^a^	66.06 ± 1.21 ^a,b^	65.89 ± 2.03 ^a,b^	66.54 ± 1.94 ^a,b^	67.62 ± 1.82 ^a^	62.48 ± 1.22 ^b^
Viability	67.37 ± 1.74 ^b,c^	77.76 ± 1.31 ^a^	73.08 ± 2.49 ^a,b^	67.54 ± 2.45 ^b,c^	67.53 ± 2.49 ^b,c^	74.54 ± 1.84 ^a^	63.99 ± 2.35 ^c^
Oxidative stress	69.52 ± 3.15 ^a^	49.92 ± 3.25 ^b^	61.78 ± 4.18 ^a^	72.22 ± 2.64 ^a^	64.64 ± 3.62 ^a^	65.09 ± 2.95 ^a^	65.93 ± 3.39 ^a^
DNA fragmentation	4.44 ± 0.60 ^b^	1.42 ± 0.16 ^c^	2.78 ± 0.58 ^b,c^	7.64 ± 0.73 ^a^	3.95 ± 0.65 ^b^	3.78 ± 0.66 ^b^	4.32 ± 0.79 ^b^

Different literals within lines mean statistical differences, *p* < 0.05. hMMP, high mitochondrial membrane potential; DNA, deoxyribonucleic acid.

## Data Availability

The original contributions presented in this study are included in the article. Further inquiries can be directed to the corresponding author(s).

## Abbreviations

The following abbreviations are used in this manuscript:

DNA	Deoxyribonucleic acid
CASA	Computer-assisted sperm analysis
MOT	Total motility
PMOT	Progressive motility
VAP	Average path velocity
VSL	Straight-line velocity
VCL	Curvilinear velocity
ALH	Amplitude of lateral head displacement
STR	Straight-line velocity
LIN	Linearity
WOB	Wobble
CFDA	Carboxyfluorescein diacetate
PI	Propidium iodide
PNA	Peanut agglutinin
DMSO	Dimethyl sulfoxide
JC-1	1,1′, 3,3′-Tetraethyl-5,5′, 6,6′-tetrachloroimidacarbocyanine iodide
SPSS	Statistical package for the social sciences
ANOVA	Analysis of variance
HMMP	High mitochondrial membrane potential
MMP	Mitochondrial membrane potential
PRP	Platelet-rich plasma
TGF-β	Transforming growth factor beta
IGF-1	Insulin-like growth factor 1
FGF	Fibroblast growth factor
VEGF	Vascular endothelial growth factor
PDGF	Platelet-derived growth factor
NGF	Nerve growth factor
SOD	Superoxide dismutase
ATP	Adenosine triphosphate
DHE	Dihydroethidium
SCSA	Sperm chromatin structure assay
dsDNA	Double-stranded DNA
ssDNA	Single-stranded DNA
SEM	Standard error of the mean

## References

[1] Gómez-Torres M.J., Medrano L., Romero A., Fernández-Colom P.J., Aizpurúa J. (2017). Effectiveness of human spermatozoa biomarkers as indicators of structural damage during cryopreservation. Cryobiology.

[2] Yeste M. (2016). Sperm cryopreservation update: Cryodamage, markers, and factors affecting the sperm freezability in pigs. Theriogenology.

[3] Hezavehei M., Sharafi M., Kouchesfahani H.M., Henkel R., Agarwal A., Esmaeili V., Shahverdi A. (2018). Sperm cryopreservation: A review on current molecular cryobiology and advanced approaches. Reprod. Biomed. Online.

[4] Sztein J.M., Noble K., Farley J.S., Mobraaten L.E. (2001). Comparison of permeating and nonpermeating cryoprotectants for mouse sperm cryopreservation. Cryobiology.

[5] Amidi F., Pazhohan A., Shabani Nashtaei M., Khodarahmian M., Nekoonam S. (2016). The role of antioxidants in sperm freezing: A review. Cell Tissue Bank..

[6] Shokri S., Ebrahimi S.M., Ziaeipour S., Nejatbakhsh R. (2019). Effect of insulin on functional parameters of human cryopreserved sperms. Cryobiology.

[7] Yang C., Xu L., Cui Y., Wu B., Liao Z. (2019). Potent humanin analogue (HNG) protects human sperm from freeze–thaw-induced damage. Cryobiology.

[8] Alcay S., Aktar A., Koca D., Kilic M.A., Akkasoglu M., Sagirkaya H. (2021). Positive effect of autologous platelet-rich plasma on Saanen buck semen cryopreservation in the non-breeding season. Cryobiology.

[9] Samadi P., Sheykhhasan M., Khoshinani H.M. (2019). The use of platelet-rich plasma in aesthetic and regenerative medicine: A comprehensive review. Aesthetic Plast. Surg..

[10] Yan B., Zhang Y., Tian S., Hu R., Wu B. (2021). Effect of autologous platelet-rich plasma on human sperm quality during cryopreservation. Cryobiology.

[11] Appel T.R., Pötzsch B., Müller J., von Lindern J.J., Bergé S.J., Reich R.H. (2002). Comparison of three different preparations of platelet concentrates for growth factor enrichment. Clin. Oral Implants Res..

[12] Marx R.E. (2004). Platelet-rich plasma: Evidence to support its use. J. Oral Maxillofac. Surg..

[13] Laškaj R., Dodig S., Čepelak I., Kuzman I. (2009). Superoxide dismutase, copper and zinc concentrations in platelet-rich plasma of pneumonia patients. Ann. Clin. Biochem..

[14] Castillo T.N., Pouliot M.A., Kim H.J., Dragoo J.L. (2011). Comparison of growth factor and platelet concentration from commercial platelet-rich plasma separation systems. Am. J. Sports Med..

[15] Magalon J., Bausset O., Serratrice N., Giraudo L., Aboudou H., Veran J., Sabatier F. (2014). Characterization and comparison of five platelet-rich plasma preparations in a single-donor model. Arthroscopy.

[16] Iyibozkurt A.C., Balcik P., Bulgurcuoglu S., Arslan B.K., Attar R., Attar E. (2009). Effect of vascular endothelial growth factor on sperm motility and survival. Reprod. Biomed. Online.

[17] Sharkey D.J., Tremellen K.P., Briggs N.E., Dekker G.A., Robertson S.A. (2016). Seminal plasma transforming growth factor-β, activin A and follistatin fluctuate within men over time. Hum. Reprod..

[18] Abdulla A.K., Rebaï T., Al-Delemi D.H.J. (2022). Protective effects of autologous platelet-rich plasma (PRP) on the outcome of cryopreservation in rabbit sperm. Mol. Cell. Biol..

[19] Li X., Wang L., Li Y., Zhao N., Zhen L., Fu J., Yang Q. (2016). Calcium regulates motility and protein phosphorylation by changing cAMP and ATP concentrations in boar sperm in vitro. Anim. Reprod. Sci..

[20] Kerns K., Zigo M., Sutovsky P. (2018). Zinc: A necessary ion for mammalian sperm fertilization competency. Int. J. Mol. Sci..

[21] Rossi T., Mazzilli F., Delfino M., Dondero F. (2001). Improved human sperm recovery using superoxide dismutase and catalase supplementation in semen cryopreservation procedures. Cell Tissue Bank..

[22] Kotdawala A.P., Kumar S., Salian S.R., Thankachan P., Govindraj K., Kumar P., Kalthur G., Adiga S.K. (2012). Addition of zinc to human ejaculate prior to cryopreservation prevents freeze–thaw-induced DNA damage and preserves sperm function. J. Assist. Reprod. Genet..

[23] Padilha R.T., Magalhães-Padilha D.M., Cavalcante M.M., Almeida A.P., Haag K.T., Gastal M.O., Nunes J.F., Rodrigues A.P.R., Figueiredo J.R., Oliveira M.A.L. (2012). Effect of insulin-like growth factor-I on some quality traits and fertility of cryopreserved ovine semen. Theriogenology.

[24] Saeednia S., Shabani Nashtaei M., Bahadoran H., Aleyasin A., Amidi F. (2016). Effect of nerve growth factor on sperm quality in asthenozoospermic men during cryopreservation. Reprod. Biol. Endocrinol..

[25] Ulhe S.M., Choudhary N., Shrivastava J., Dutta S., Dakre S.M., More A. (2024). Overcoming male factor infertility: A journey through assisted reproductive technology with platelet-rich plasma therapy. Cureus.

[26] Saucedo L., Buffa G.N., Rosso M., Guillardoy T., Góngora A., Munuce M.J., Vazquez-Levin M.H., Marín-Briggiler C. (2015). Fibroblast growth factor receptors (FGFRs) in human sperm: Expression, functionality and involvement in motility regulation. PLoS ONE.

[27] Lorian K., Haghdani S., Vahidi S., Nabi A. (2024). Application of autologous platelet-rich plasma exerts cryoprotective effects on biological characteristics of human oligoasthenoteratospermia samples after freezing and thawing procedures. Urol. J..

[28] Jiménez-Trejo F., Tapia-Rodríguez M., Cerbón M., Kuhn D.M., Manjarrez-Gutiérrez G., Mendoza-Rodríguez C.A., Picazo O. (2012). Evidence of 5-HT components in human sperm: Implications for protein tyrosine phosphorylation and the physiology of motility. Reproduction.

[29] Moradian S.A., Amirkhani Z., Movahedin M., Gharib S.N., Varghaiyan Y., Niknafs B., Hamdi K., Hajipour H., Fattahi A. (2025). Platelet-rich plasma (PRP) and the future of male fertility: A path forward for personalized and regenerative therapies. Stem Cell Res. Ther..

[30] Hamdan D.A., Rahim A.I., Al-Kawaz U.M. (2021). In vitro studying the effect of adding autologous platelet-rich plasma (PRP) to human semen on sperm DNA integrity. Iraqi J. Embryos Infertil. Res..

[31] Ghasemian Nafchi H., Azizi Y., Amjadi F., Halvaei I. (2023). In vitro effects of plasma rich in growth factors on human teratozoospermic semen samples. Syst. Biol. Reprod. Med..

[32] Nabavinia M.S., Yari A., Ghasemi-Esmailabad S., Gholoobi A., Gholizadeh L., Nabi A., Lotfi M., Khalili M.A. (2023). Improvement of human sperm properties with platelet-rich plasma as a cryoprotectant supplement. Cell Tissue Bank..

[33] Mariano R., Messora M., de Morais A., Nagata M., Furlaneto F., Avelino C., Paula F., Ferreira S., Pinheiro M., de Sene J.P. (2010). Bone healing in critical-size defects treated with platelet-rich plasma: A histologic and histometric study in the calvaria of diabetic rat. Oral Surg. Oral Med. Oral Pathol. Oral Radiol. Endod..

[34] Ungerfeld R., Viera M.N., Freitas-de-Melo A., Giriboni J., Casuriaga D., Silveira P. (2021). Seasonality of the stress response in goat bucks when electroejaculation is used for semen collection. Anim. Reprod. Sci..

[35] Toker M., Alcay S. (2022). Comprehensive effects of fetal calf serum in soybean-lecithin-based goat semen cryopreservation extenders and impacts on incubation resilience. Kafkas Univ. Vet. Fak. Derg..

[36] Tekin K., Daşkın A. (2016). Effect of different extenders on motility and some sperm kinematics parameters in Norduz goat semen. Turk. J. Vet. Anim. Sci..

[37] Câmara D.R., Silva S.V., Almeida F.C., Nunes J.F., Guerra M.M.P. (2011). Effects of antioxidants and duration of pre-freezing equilibration on frozen-thawed ram semen. Theriogenology.

[38] Marco-Jiménez F., Puchades S., Gadea J., Vicente J.S., Viudes-de-Castro M.P. (2005). Effect of semen collection method on pre- and post-thaw Guirra ram spermatozoa. Theriogenology.

[39] Dariush G., Gholamhossein R., Rouhollah F., Seyed Mahmood G., Abdolhossein S., Mohsen S., Loghman A. (2019). The application of ultrasonic vibration in human sperm cryopreservation as a novel method for the modification of physicochemical characteristics of freezing media. Sci. Rep..

[40] Peña F.J., Ball B.A., Squires E.L. (2018). A new method for evaluating stallion sperm viability and mitochondrial membrane potential in fixed semen samples. Cytom. B Clin. Cytom..

[41] Eser A., Yağcıoğlu S., Arıcı R., Demir K., Ak K. (2024). Effects of resveratrol-loaded cyclodextrin on the quality characteristics of ram spermatozoa following cryopreservation. Animals.

[42] Evenson D.P., Larson K.L., Jost L.K. (2002). Sperm chromatin structure andrology lab corner assay: Its clinical use for detecting sperm DNA fragmentation in male infertility and comparisons with other techniques. J. Androl..

[43] Sharma A., Sood P. (2020). Caprine semen cryopreservation and the factors affecting it: An overview. Vet. Sci. Res. Rev..

[44] Oktanella Y., Mustofa I., An-Haru F.A.R., Putri D.D.M., Hendrawan V.F., Susilowati S., Degu N.Y., Hernawati T. (2024). Conserving goat sperm post-thawed gene expression and cellular characteristics using the antioxidant coenzyme Q10 supplementation. Vet. World.

[45] Khương T.T.T., Duy N.L.K., Nhi L.T.Y. (2024). Effects of cysteine on goat sperm quality in cryopreservation. Vietnam J. Biotechnol..

[46] Güngör Ş., İnanç M.E., Uslu B.A., Burca A.B., Ata A. (2025). Impact of Trolox supplementation on the cryopreservation of Honamli buck semen. Biopreserv. Biobank..

[47] Salama M.S., Shehabeldin A.M., Ashour M.A., Al-Ghadi M.Q., Marghani B.H., El-kon I., Shukry M. (2024). Effect of the addition of platelet-rich plasma to Boer buck semen on sperm quality and antioxidant activity before and after cryopreservation and in vivo fertility. Small Rumin. Res..

[48] Almadaly E.A., Mansour I., Sahwan F., Shehabeldin A.M., El-Kon I., Sakr A., Eldomany W., Ramoun A.A. (2024). Effect of adding autologous platelet-rich plasma to the freezing extender on the post-thaw quality and in vitro fertility of buffalo (Bubalus bubalis) spermatozoa. Egypt. J. Vet. Sci..

[49] Pang K.H. (2025). The role and implication of platelet-rich plasma in male factor infertility: A systematic review of human studies. Andrology.

[50] Troha K., Vozel D., Battelino S. (2023). Storage of platelet-rich products. Proc. Socrat. Lect..

[51] Güngör İ.H., Türk G., Çakır Cihangiroğlu A., Arkalı G., Tektemur A., Kırmızıkaya Özmen G., Badıllı N., Acısu T.C., Bulan M.S., Özer Kaya Ş. (2025). Platelet-rich plasma strategy against freezing damage in ram spermatozoa: Its effect on miRNA, ion channels, growth factors, lipids and oxidative stress. Reprod. Fertil. Dev..

[52] Salama M.S., Ashour M.A., Taher E.S., Rashed F., Ibrahim I.M., El-Nablaway M., Megahed Ibrahim A., Mihaela O., Olga R., Mohammed N.A. (2024). Effect of autologous platelet-rich plasma on the fertility and quality of cryopreserved buffalo bull semen: A comparative study using OptiXcell^®^ and tris egg yolk extenders. BMC Vet. Res..

[53] Hackerova L., Pilsova A., Pilsova Z., Zelenkova N., Tymich Hegrova P., Klusackova B., Chmelikova E., Sedmikova M., Simonik O., Postlerova P. (2025). Boar Sperm Motility Assessment Using Computer-Assisted Sperm Analysis: Current Practices, Limitations, and Methodological Challenges. Animals.

[54] Ohtani K., Yamazaki S., Kubota H., Miyagawa M., Saegusa J. (2004). Comparative investigation of several sperm analysis methods for evaluation of spermatotoxicity of industrial chemical: 2-bromopropane as an example. Ind. Health.

[55] Rajashri M., Reddy K.R., Kumari G.A., Kumari N.N., Srinivas G. (2018). Computer assisted semen analysis of Deccani ram semen preservability at 5 °C. Indian J. Anim. Res..

[56] Alquézar-Baeta C., Gimeno-Martos S., Miguel-Jiménez S., Santolaria P., Yániz J., Palacín I., Casao A., Cebrián-Pérez J.Á., Muiño-Blanco T., Pérez-Pé R. (2019). OpenCASA: A new open-source and scalable tool for sperm quality analysis. PLoS Comput. Biol..

[57] Zadmajid V., Myers J.N., Sørensen S.R., Butts I.A. (2019). Ovarian fluid and its impacts on spermatozoa performance in fish: A review. Theriogenology.

[58] El-Sherbiny H.R., Abdelnaby E.A., Samir H., Fathi M. (2022). Addition of autologous platelet rich plasma to semen extender enhances cryotolerance and fertilizing capacity of buffalo bull spermatozoa. Theriogenology.

[59] Alcay S., Aktar A., Koca D., Kilic M.A., Akkasoglu M., Yilmaz M.M., Sagirkaya H. (2022). Autologous platelet rich plasma have positive effect on ram spermatozoa during cryopreservation in non-breeding season. Kafkas Univ. Vet. Fak. Derg..

[60] Bader R., Ibrahim J.N., Moussa M., Mourad A., Azoury J., Alaaeddine N. (2020). In vitro effect of autologous platelet-rich plasma on H_2_O_2_-induced oxidative stress in human spermatozoa. Andrology.

[61] Angellee J., Ginting C.N., Chiuman L., Halim B. Role of platelet-rich plasma to sperm quality in male partners undergoing infertility treatment. Proceedings of the 2021 IEEE International Conference on Health, Instrumentation & Measurement, and Natural Sciences (InHeNce).

[62] Quintana F., Vendrell A., Fernandez S.P., Fuente M., Merino-Pérez A., Ferrando M., Matorras R. (2024). Safety of plasma rich in growth factors (PRGF) as additive to healthy human sperm samples: A pilot study. JBRA Assist. Reprod..

[63] Mirzaei J., Movahedin M., Halvaei I. (2022). Plasma-rich in growth factors ameliorates detrimental effects of cryopreservation on human sperm: A prospective study. Cell J. (Yakhteh).

[64] Almadaly E.A., Ibrahim I.M., Salama M.S., Ashour M.A., Sahwan F.M., El-Kon I.I., Abouzed T.K., El-Domany W.B. (2023). Effect of platelet-rich plasma (PRP) on post-thaw quality, kinematics and in vivo fertility of fertile and subfertile buffalo (Bubalus bubalis) spermatozoa. Vet. Res. Commun..

[65] Niżański W., Partyka A., Prochowska S. (2016). Evaluation of spermatozoal function—Useful tools or just science. Reprod. Domest. Anim..

[66] Šutovský P., Aarabi M., Miranda–Vizuete A., Oko R. (2015). Negative biomarker based male fertility evaluation: Sperm phenotypes associated with molecular-level anomalies. Asian J. Androl..

[67] Kooli R., Boussabbeh M., Chebil D., Kenani A., Khefacha L., Mehdi M., Sallem A. (2025). Platelet-rich plasma: A promising therapy for mitigating sperm oxidative stress and mitochondrial dysfunction in subfertile men. PLoS ONE.

[68] Zhang W.-D., Zhang Z., Jia L.-T., Zhang L.-L., Fu T., Li Y.-S., Wang P., Sun L., Shi Y., Zhang H.-Z. (2014). Oxygen free radicals and mitochondrial signaling in oligospermia and asthenospermia. Mol. Med. Rep..

[69] Soltani M., Shojafar E., Ghafarizadeh A.A., Moslemi A., Mashayekhi F.J., Baazm M. (2025). Comparison of the effects of platelet-rich plasma and nanocurcumin on the sperm quality parameters in frozen-thawed semen of men with asthenoteratozoospermia: A lab trial study. Int. J. Reprod. Biomed..

[70] Mughal D.H., Ijaz A., Yousaf M.S., Wadood F., Farooq U., Mahmood S.A., Riaz A. (2018). Effect of osmotic pressure on spermatozoa characteristics of cryopreserved buffalo bull (*Bubalus bubalis*) semen. J. Appl. Anim. Res..

